# T123. PERSISTENT NEGATIVE SYMPTOMS IN FIRST EPISODE PSYCHOSIS: PREVALENCE, PREDICTORS AND PROGNOSIS

**DOI:** 10.1093/schbul/sby016.399

**Published:** 2018-04-01

**Authors:** Stephen Austin, Carsten Hjorthøj, Ole Mors, Rikke Gry Secher, Pia Jeppesen, Lone Petersen, Anne Thorup, Merete Nordentoft

**Affiliations:** 1 Psychiatric Research Unit; 2 Copenhagen University Hospital, Mental Health Center Copenhagen; 3 Aarhus University Hospital; 4 University of Copenhagen; 5 Child and Adolescent Mental Health Center

## Abstract

**Background:**

Negative symptoms are a core component of schizophrenia. These symptoms have been shown to impact on a range of outcomes, and often are resistant to pharmacological and psychosocial interventions treatment. The goal of this study was to investigate the prevalence, baseline predictors and long-term impact of persistent negative symptoms (PNS) within a large representative cohort of people with first episode psychosis.

**Methods:**

The study had prospective design. Patients recruited into the OPUS trial (1998–2000) with a first time diagnosis within the schizophrenia spectrum (F20-28) were included. People were classified with persistent negative symptoms, if they experienced enduring negative symptoms that were not secondary to psychotic symptoms, depression or due to medication side effects. Clinical data collected at baseline, 1 year, 2 years and 10 years was used to identify predictors of PNS and long-term outcomes.

**Results:**

Full clinical data was available on 369 people. A total of 90 people (24%) displayed PNS, two years after diagnosis. Significant univariable predictors of PNS at baseline were low functioning, male sex, cannabis use, poor pre-morbid social functioning and high levels of negative symptoms. People that displayed PNS had significantly lower functioning and higher levels of psychopathology at 10 year follow-up. A total 3% of people with PNS were recovered at 10 year follow-up compared to rate of 20% recovered without PNS (OR 7.42, p<0.01).

**Discussion:**

A significant proportion of the cohort displayed persistent negative symptoms and these symptoms significantly impacted on long-term outcomes. Researchers and clinicians need to continue to develop effective interventions that can ameliorate these symptoms and potentially impact on illness prognosis within schizophrenia.

